# The Pathogenesis of COVID-19 Myocardial Injury: An Immunohistochemical Study of Postmortem Biopsies

**DOI:** 10.3389/fimmu.2021.748417

**Published:** 2021-11-05

**Authors:** Camila Hartmann, Anna Flavia Ribeiro dos Santos Miggiolaro, Jarbas da Silva Motta, Lucas Baena Carstens, Caroline Busatta Vaz De Paula, Sarah Fagundes Grobe, Larissa Hermann de Souza Nunes, Gustavo Lenci Marques, Peter Libby, Lidia Zytynski Moura, Lucia de Noronha, Cristina Pellegrino Baena

**Affiliations:** ^1^School of Medicine, Pontifícia Universidade Católica do Paraná (PUCPR), Curitiba, Brazil; ^2^Department of Medicine, Marcelino Champagnat Hospital, Curitiba, Brazil; ^3^Division of Cardiovascular Medicine, Department of Medicine, Brigham and Women’s Hospital, Harvard Medical School, Boston, MA, United States

**Keywords:** heart, myocardial injury, COVID-19, SARS-CoV-2, endothelium, immunohistochemistry, pathology, fibrosis

## Abstract

**Rationale:**

Myocardial injury associates significantly and independently with mortality in COVID-19 patients. However, the pathogenesis of myocardial injury in COVID-19 remains unclear, and cardiac involvement by SARS-CoV-2 presents a major challenge worldwide.

**Objective:**

This histological and immunohistochemical study sought to clarify the pathogenesis and propose a mechanism with pathways involved in COVID-19 myocardial injury.

**Methods and Results:**

Postmortem minimally invasive autopsies were performed in six patients who died from COVID-19, and the myocardium samples were compared to a control group (n=11). Histological analysis was performed using hematoxylin-eosin and toluidine blue staining. Immunohistochemical (IHC) staining was performed using monoclonal antibodies against targets: caspase-1, caspase-9, gasdermin-d, ICAM-1, IL-1β, IL-4, IL-6, CD163, TNF-α, TGF-β, MMP-9, type 1 and type 3 collagen. The samples were also assessed for apoptotic cells by TUNEL. Histological analysis showed severe pericardiocyte interstitial edema and higher mast cells counts per high-power field in all COVID-19 myocardium samples. The IHC analysis showed increased expression of caspase-1, ICAM-1, IL-1β, IL-6, MMP-9, TNF-α, and other markers in the hearts of COVID-19 patients. Expression of caspase-9 did not differ from the controls, while gasdermin-d expression was less. The TUNEL assay was positive in all the COVID-19 samples supporting endothelial apoptosis.

**Conclusions:**

The pathogenesis of COVID-19 myocardial injury does not seem to relate to primary myocardiocyte involvement but to local inflammation with associated interstitial edema. We found heightened TGF-β and interstitial collagen expression in COVID-affected hearts, a potential harbinger of chronic myocardial fibrosis. These results suggest a need for continued clinical surveillance of patients for myocardial dysfunction and arrythmias after recovery from the acute phase of COVID-19.

## Introduction

Since its emergence in December 2019 (1), the coronavirus disease 2019 (COVID-19) pandemic, caused by severe acute respiratory syndrome coronavirus 2 (SARS-CoV-2), continues to grow despite unprecedented worldwide efforts in the search of treatments and vaccines. COVID-19 is initially a respiratory disease, causing viral pneumonia and adult respiratory distress syndrome. However, cardiovascular manifestations occur commonly and relate to poor outcomes (2).

Myocardial injury (defined by troponin blood levels above the 99th-percentile upper reference) was observed in 7 to 17% (3) of patients and associates significantly and independently with mortality (4). Common cardiac complications among hospitalized patients with COVID-19 include arrhythmias and acute heart failure. Heart failure may contribute up to 40% of deaths, and circulatory failure may cause death even without respiratory failure (3). A prothrombotic coagulopathy can occur in 25% of patients resulting in venous and arterial thromboembolic events (5).

SARS-CoV-2 genome detection in endomyocardial biopsies (6) and autopsy specimens (7) has localized SARS-CoV-2 in the heart. However, evidence for infection of cardiocytes themselves remains controversial and the mechanism of cardiac damage by SARS-CoV-2 remains incompletely understood. Some autopsies of patients with COVID-19 revealed accumulation in the myocardial interstitium of mononuclear inflammatory cells (8) while others showed no increase in inflammatory cells despite the presence of the viral genome (7). SARS-CoV-2 particles have already been observed in myocardial interstitial cells (9) and endothelial cells (10) by electron microscopy, and it has been proposed that pyroptosis may have an important role in endothelial cell injury in patients with COVID-19 (10). Pyroptosis is a specific type of programmed pro-inflammatory cell death that culminates in caspase-1 activation, interleukin-6 (IL-6) secretion and endothelial disfunction (11). This could be the initial pathway for myocardial injury, and could also explain the involvement of various organs and tissues that has been described in COVID-19.

Interstitial myocardial fibrosis has been described as a possible consequence of myocardial injury (6, 12) by the expression of the pro-fibrotic mediator TGF-β, which can stimulate interstitial collagen production (13). Myocardial fibrosis may predispose to both systolic and diastolic dysfunction, as well as arrhythmias (14).

Given that the cardiac manifestations play a major role in adverse outcomes and discordant pathological studies regarding the mechanism of myocardial injury in COVID-19, we investigated myocardium samples in a histological and immunohistochemical study to help clarify its pathogenesis in lethal cases. Our findings provide new insight into the mechanisms involved in COVID-19-related myocardial injury.

## Methods

Postmortem minimally invasive autopsies were performed in six patients who died from COVID-19 in Marcelino Champagnat Hospital, Brazil. All patients were symptomatic and tested positive for SARS-CoV-2 on nasopharyngeal swabs (RT-PCR) and had the chest computed tomography at admission compatible with pulmonary infection by COVID-19. This study was approved by the National Research Ethics Committee (CONEP), protocol number 3.944.734/2020. Patients’ families authorized the autopsies and provided informed consent form before the procedures. All methods were carried out following relevant guidelines and regulations. Clinical data were obtained from medical records during hospitalization in the Intensive Care Unit (ICU).

Myocardial tissue was collected within 4 hours after death by left anterior mini thoracotomy for direct access to the left ventricle. The pericardium was sectioned and a fragment of myocardial tissue approximately 2 x 2 cm was obtained. The tissues from the myocardial biopsies were fixed in neutral buffered formalin for over 24 hours, and then processed under conventional histological technique. The myocardium biopsies of patients with COVID-19 were then independently compared to myocardium samples from control patients 6 hours after death from other. All the results were analyzed and integrated to the previous pathological knowledge.

### Histological and Immunohistochemical Analysis

The formalin fixed paraffin embedded (FFPE) sections were subjected to hematoxylin-eosin (H&E) and toluidine blue (TB) staining. Histological features (H&E) were observed and described by using Olympus BX40. Mast cells (only nucleated cells with granules) were scored (TB) by counting cells per high-power field (HPF – 40x objective – 0.26mm^2^) by screening 20 randomized HPFs (total area of 5.2mm^2^ per case).

Immunohistochemical (IHC) staining was performed in the myocardium samples using monoclonal and polyclonal antibodies against the following targets: caspase-1, caspase-9, gasdermin-d and interleukin-1β (IL-1β) to detect pyroptosis; intercellular adhesion molecule-1 (ICAM-1), tumor necrosis factor alpha (TNF-α), interleukin-4 (IL-4), interleukin-6 (IL-6), CD163 (macrophage-specific protein) and matrix metalloproteinase-9 (MMP-9) to detect inflammatory activation and response pathways; transforming growth factor (TGF-β), type 1 and type 3 collagen to detect myocardial fibrosis. The table in the **Supplementary Material** summarizes the specifications of the antibodies used to investigate the FFPE myocardial tissues. In order to detect apoptosis, the samples were also subjected to a TUNEL assay (Terminal deoxynucleotidyl transferase dUTP nick end labeling), using the ‘*In Situ* Cell Death Detection Kit, POD’ by Roche.

Scores of biomarker expression according to the IHC staining were given by an experienced pathologist and confirmed by two trained technicians. Biomarkers were analyzed using Allred scoring system (15, 16): score 0-5 depending on the proportion of cells which are stained (proportion score [PS]: score 0 = none stained cells, score 1 = 1%, score 2 = 1-10%, score 3 = 11-33%, score 4 = 34-66% and score 5 = 66-100%) and score 0-3 depending on the intensity of staining (intensity score [IS]: score 0 = none stained cells, score 1 = weak, score 2 = moderate, score 3 = strong). The semiquantitative analysis was obtained by summing the two scores (proportion and intensity of positivity), ranging from 0 to 8.

### Statistical Analysis

Clinical data and biomarkers score expression were evaluated independently in COVID-19 patients and controls. The comparison of the quantitative variables of the two groups was performed using the non-parametric Mann-Whitney test and the comparison of the proportions between groups was performed with Chi-Square. Values of p < 0.05 indicated statistical significance. The data were analyzed using the GraphPad Prism v9.0.2 software.

## Results

### Patient Clinical Data

Clinical data from the baseline of COVID-19 patients (n=6) and control group (n=11) are presented in **Table 1**. Our sample was mainly male, with median age of 73.5 years, hypertensive, diabetic, with history of coronary artery disease and with no significant difference between groups (p>0.05 for all). Causes of death in the control group include bronchopneumonia (n=3), pulmonary thromboembolism (n=3), mesenteric ischemia (n=2), myocardial infarction (n=1), aortic dissection (n=1) and upper gastrointestinal bleeding (n=1).

**Table 1:** Clinical data from the baseline of COVID-19 and control patients.

	COVID-19 (n = 6)	Controls (n = 11)
**Sex (male)**	5	7
**Age (median)**	74	73
**Former smoker**	1	6
**Hypertension**	5	10
**Diabetes mellitus**	5	8
**Coronary artery disease**	5	9
**Heart failure**	3	6
**Cancer**	0	2
**Chronic pulmonary disease**	1	6
**Chronic kidney disease**	1	3
**Length of stay on Mechanical Ventilation (median)**	12 days	–

All the COVID-19 patients had symptoms of dyspnea with progressive worsening and had the chest computed tomography at admission suggestive of viral pulmonary infection for COVID-19. They were all admitted into the ICU and developed respiratory failure requiring mechanical ventilation. The median duration of mechanical ventilation was 12 days. During hospitalization, three patients developed acute kidney failure and patient 1 had pre-existing dialysis-dependent chronic kidney disease; two patients had incident acute atrial fibrillation; and one patient had an acute pulmonary embolism. Laboratory evaluation showed high levels of D-dimer and troponin in all COVID-19 patients. Transthoracic echocardiographic findings were heterogeneous as described in the **Supplementary Material**. Patients 2 and 5 had normal echocardiographic results with normal ejection fraction.

### Histological Analysis

The sample tissues from the COVID-19 patients were compared to the control group samples and histological assessment showed severe pericellular interstitial edema surrounding each cardiomyocyte in all of the COVID-19 patients. Histological analysis also showed neutrophilic myocarditis according to the Dallas criteria (17) in patient 1. All the other COVID-19 samples showed neither massive inflammatory cellular infiltration nor necrosis, indicating the absence of typical histological myocarditis. In contrast, toluidine blue staining revealed a higher interstitial and perivascular mast cell score in all COVID-19 myocardium samples compared to control (p=0.0023). Most mast cells appeared to be degranulating. Lipofuscin pigment and mild signs of cardiomyocyte hypertrophy were seen in COVID-19 and control patients.

### Immunohistochemical Analysis

**Figure 1:**
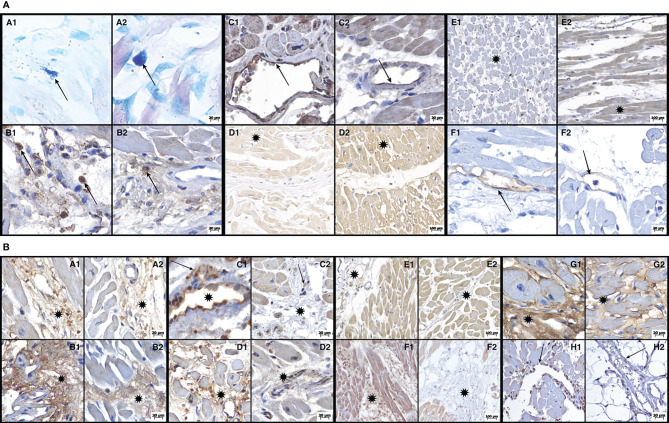
**(A)** Photomicrographs demonstrating histochemical (A1/2 - Toluidine Blue) and immunohistochemical (B = CD163; C = Casp-1; D = Casp-9; E = GSDM-D; F = ICAM-1) reactions of both groups: cases of COVID-19 (B1-F1) and cases of their respective controls (B2-F2). A1 (COVID-19) and A2 (control) show a mast cell in the perivascular space (arrows): in A1 the mast cell is degranulated, whereas in A2, the mast cell is intact. B1 (COVID-19) shows CD163-immunostained macrophages (arrows) in the myocardial interstitium, whereas in B2 (control) these CD163 macrophages (arrow) are less numerous. C1 (COVID-19) shows endothelial cells (arrow) with strong expression of Casp-1, whereas in C2 (control), Casp-1 expression in endothelial cells (arrow) is much more discrete. D1 (COVID-19) shows a weak expression of Casp-9 in the myocardium (asterisk), whereas in D2 (control), this expression is strong (asterisk). E1 (COVID-19) shows a weak expression of GSDM-D in the myocardium (asterisk), with E2 (control) showing a strong expression (asterisk). F1 (COVID-19) shows a strong expression of ICAM-1 in endothelial cells (arrow), whereas, in F2 (control), this expression is discrete (arrow). **(B)** Photomicrographs demonstrating immunohistochemical reactions (A = TNF-α; B = MMP-9; C = IL-4; D = IL-6; E = IL-1β; F = TGF-β; G = collagen 3; H = TUNEL) from both groups: cases of COVID-19 (A1-H1) and their respective controls (A2-H2). A1 (COVID-19) shows myocardial interstitium expressing TNF-α (asterisk), whereas, in A2 (control), we can observe that this expression is discrete (asterisk). B1(COVID-19) shows myocardial interstitium expressing MMP-9 (asterisk), whereas, in B2 (control), this expression is discrete (asterisk). C1 (COVID-19) shows interstitial macrophages (arrow) and endothelial cells (asterisk) with strong expression of IL-4, whereas in C2 (control), the expression of IL-4 in interstitial macrophages (arrow) and endothelial cells (asterisk) is discrete. D1 (COVID-19) shows a strong expression of IL-6 in the interstitium (asterisk), whereas, in D2 (control), this expression is discrete (asterisk). E1 (COVID-19) shows moderate expression of IL-1β in the myocardial interstitium (asterisk), and E2 (control) has a more discrete expression (asterisk). F1 (COVID-19) shows a strong expression of TGF-β in the interstitium of the myocardium (asterisk), whereas, in F2 (control), this expression is much more discrete or absent (asterisk). G1 (COVID-19) shows a strong expression of collagen 3 in the interstitium of the myocardium (asterisk), and this expression is much more delicate (asterisk) in G2 (control). H1 (COVID-19) demonstrates elevated DNA fragmentation in the TUNEL assay (arrow) compared to H2 (control), where few nuclei (arrow) are labeled.

Images of H&E, TB and IHC staining for COVID-19 patients and controls are shown in **Figures 1A, B**. The full scores of biomarker expression according to the IHC analysis are shown in the **Supplementary Material**.

**Figure 2:**
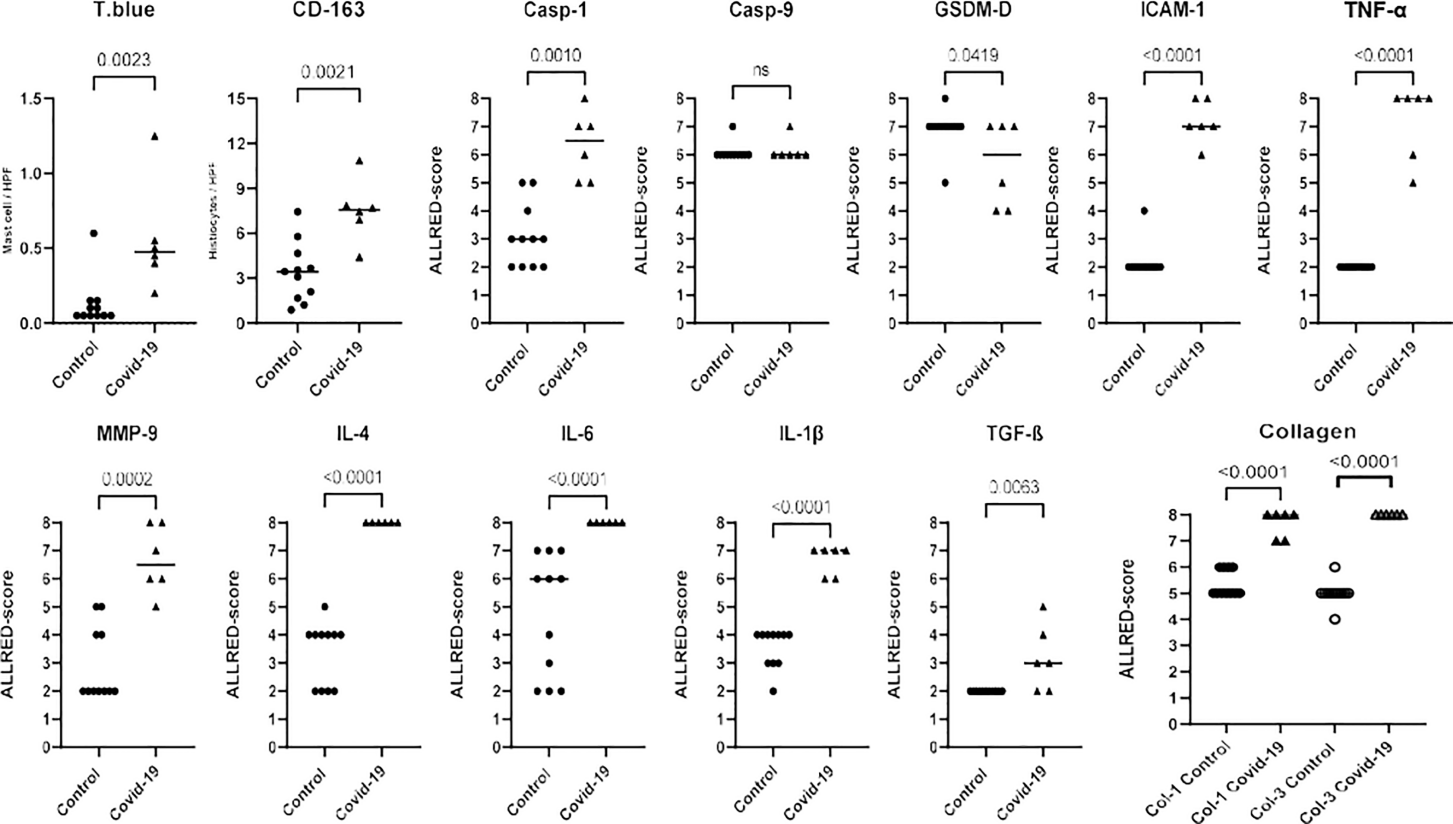
Graphical representation of the immunohistochemical analysis according to the biomarker expression (Toluidine Blue; CD-163; Casp-1; Casp-9; GSDM-D; ICAM-1; TNF-α; MMP-9; IL-4; IL-6; IL-1β; TGF-β; Collagen 1 and 3) in COVID 19 cases and controls. Biomarkers were analyzed using Allred scoring system: score 0-5 depending on the proportion of cells which are stained (proportion score [PS]: score 0 = none stained cells, score 1 = 1%, score 2 = 1-10%, score 3 = 11-33%, score 4 = 34-66% and score 5 = 66-100%) and score 0-3 depending on the intensity of staining (intensity score [IS]: score 0 = none stained cells, score 1 = weak, score 2 = moderate, score 3 = strong). The semiquantitative analysis was obtained by summing the two scores (proportion and intensity of positivity), ranging from 0 to 8.

The IHC analysis showed increased expression for caspase-1 (p=0.0010), ICAM (p<0.0001), CD163 (p=0.0021), IL-1β (p<0.0001), IL-4 (p<0.0001), IL-6 (p<0.0001), MMP-9 (p=0.0002), TNF-α (p<0.0001), TGF-β (p=0.0063), type 1 collagen (p<0.0001) and type 3 collagen (p<0.0001) in the COVID-19 patients compared to the control. The expression for gadesmin-d, on the other hand, was decreased (p=0.0419). The graphical representation of these analyses are shown in **Figure 2**. No substantial differences from the control were observed in expression of caspase-9 (p>0.9999).

**Figure 3:**
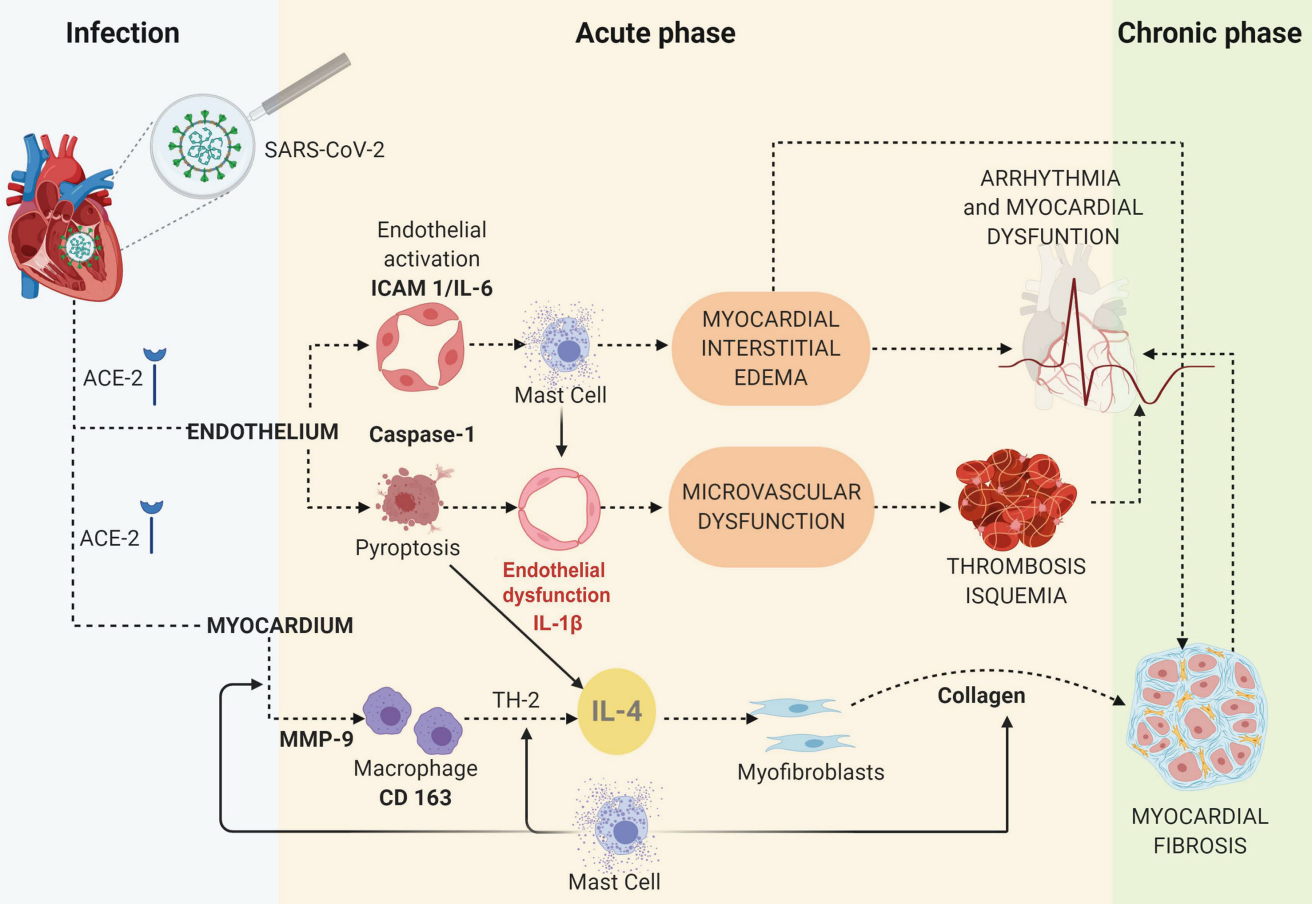
The proposed mechanisms of myocardial injury based on an IHC study of COVID-19 cases and controls.

The TUNEL assay in COVID-19 myocardium samples indicated endothelial cell apoptosis, distinct from the control samples, which tested negative. Cardiomyocytes did not display this marker of apoptosis. Other morphologic findings merit noting. First, caspase-1 and IL-6 were present in the cytoplasm, whereas ICAM-1 localized on the membrane of endothelial cells. Secondly, MMP-9 and both types of collagen were observed in large quantities in the interstitial and perivascular spaces. Our proposed mechanism of COVID-19 myocardial injury is shown in **Figure 3**.

## Discussion

Our main findings from the myocardial postmortem biopsies of COVID-19 patients show myocardial interstitial edema, mast cell accumulation, and increased indicators of inflammation, apoptosis, and fibrosis compared to controls. The increased expression of ICAM-1 and IL-6 indicates inflammatory activation (18), which along with higher mast cell scores can explain the increased capillary permeability, microvascular leakage and the consequent formation of myocardial interstitial edema (19, 20). The increased expression of MMP-9, CD163, IL-4 and IL-6 demonstrates the presence of myocardial inflammatory response in the myocardial tissue (18, 21). More specifically, CD163 indicates macrophage recruitment (21) and MMP-9 promotes Th2 cell recruitment and matrix remodeling (22). The Th2 cytokine IL-4 and TGF-β can drive myocardial fibrosis (23) and provide a mechanism of elevated interstitial collagens type 1 and type 3 (24–27).

The TUNEL positivity in all the COVID-19 samples shows that this disease promotes endothelial cell apoptosis (programmed cell death). The probable mechanism is by pyroptosis, a specific inflammatory form of apoptosis that occurs most frequently upon infection by intracellular pathogens (like SARS-CoV-2) and requires the enzyme caspase-1 (28). Caspase-1 is activated as part of a multiprotein signaling platform, the inflammasome complex, and subsequently mediates the activation and secretion of interleukins, including IL-1β, as well as the rupture of the cell membrane (29). The lack of change in caspase-9 expression and the decrease in gasdermin-d expression corroborate the inflammasome complex, since gasdermin-d is activated and cleaved in this pathway, which has interaction with caspase-1 but usually no interaction with caspase-9 (30–32).

We also observed higher levels of caspase-1 adjacent to endothelial cells in the COVID-19 samples demonstrating endothelial infection, pyroptosis and injury in these patients. Moreover, SARS-CoV-2 particles have been described in endothelial cells by electron microscopy (10) and the caspase-1 identification is in accordance with Varga et al. (10), who suggested that pyroptosis might have an important role in endothelial cell injury in patients with COVID-19. These findings are also in line with previous biopsy studies which had already shown that the inflammatory process in cardiac tissue permeates the vascular wall (6, 11). SARS-CoV-2 potentially causes endotheliitis (10), which is determinant of microvascular dysfunction by shifting the vascular equilibrium towards more vasoconstriction with subsequent organ ischemia, inflammation with associated tissue edema, and a procoagulant state (33).

The expression of IL-6 and ICAM-1 increased in the endothelial cells and indicates endothelial activation as well as immune cell recruitment and response. Activated endothelial cells translocate NF-κB p50/p65 dimers to the nucleus and induce the expression of IL-6, TNF-α, and adhesion molecules such as ICAM-1. These molecules recruit leukocytes to the infected region of COVID-19 myocardial injury and propagate inflammation (18).

Endothelial cell functions regulate local vascular permeability and blood flow (19, 33). At rest, the endothelium is highly impermeable to large molecules. However, acute changes in vascular permeability result in loss of fluid and plasma proteins from the intravascular space into the interstitium, leading to edema (19, 20, 33). The abundance of mast cells rich in histamine suggests a mechanism for vascular leakage and interstitial myocardial edema observed here in specimens from COVID-19 patients. In addition to histamine, mast cell release TNF-α and proteases, which contribute to increased vascular permeability and local inflammation (19, 20). Mast cell activation occurs not only in the context of allergy, but also in viral infection (34).

A previous autopsy study showed that the presence of SARS-CoV-2 genome in the myocardial tissue was not associated with increased infiltration of mononuclear cells compared with the virus negative group (7). Although most of our COVID-19 myocardium samples also showed neither inflammatory cellular infiltration nor necrosis, which would be expected in typical histological myocarditis, the high levels of MMP-9, CD163, IL-4 and IL-6 demonstrate the presence of myocardial inflammatory response in this tissue.

We observed elevated type 1 and type 3 collagen in the interstitial and perivascular spaces in the COVID-19 samples, suggesting myocardial fibrosis, since synthesis of both types of collagen is markedly increased in the remodeling fibrotic heart, regardless of the etiology of fibrosis (23). TGF-β has major roles in cardiac fibrogenesis (23, 27, 35) and acts by activating SMAD2/3 pathways. IL-6 and IL-4 are also profibrotic cytokines, as they induce MMP-9 expression and collagen synthesis through gene transcription modulation (24–26). Mast cell degranulation may also be involved in fibrogenesis (23), since mast cell tryptase can directly induce fibroblast activation, myofibroblast differentiation and collagen synthesis independently of TGF-β (34)

Taken together our findings indicate that the microvascular dysfunction may lead to thrombosis and underscores the need for rigorous randomized evaluation of anticoagulant and anti-aggregating therapies in various stages of COVID-19 (36). The myocardial interstitial edema observed here may contribute to the high prevalence of cardiac arrhythmia in COVID-19 patients by loss of structure of the syncytium (19). Furthermore, our findings suggest that COVID-19 myocardial injury may cause myocardial fibrosis in the long term. Therapies which act on cardiac remodeling, such as angiotensin-converting enzyme inhibitors or mineralocorticoid receptor antagonists merit evaluation for myocardial protection in patients with or recovered from the acute phase of COVID-19 (37, 38).

Some limitations should be considered in our study. Our sample size was limited, since ethical issues, labor shortages and hospital overcrowding, among many other reasons, prevented larger samples of postmortem myocardial biopsies from being collected in the COVID-19 Intensive Care Units. Therefore, it is important to interpret our findings with caution and validate them in other samples to replicate our results. Additionally, interpretation of our findings should take into account that data based on FFPE postmortem samples only provides information at the time of death, and cannot reconstruct the entire disease process.

In conclusion, our observations provide new insight into the multifactorial mechanisms that provoke myocardial injury in COVID-19 including interstitial edema associated with mast cells, local inflammation, and fibrogenesis. These findings help to understand the acute cardiac complications of COVID-19, and also raise concerns about possible chronic cardiac sequelae of SARS-CoV-2 infection which will require further study in survivors of this pandemic illness.

## Data Availability Statement

The original contributions presented in the study are included in the article/**Supplementary Material**. Further inquiries can be directed to the corresponding author.

## Ethics Statement

This study was reviewed and approved by the National Research Ethics Committee (CONEP), Pontifícia Universidade Católica do Paraná (PUCPR), protocol number 3.944.734/2020. Patients’ families authorized the autopsies and provided written informed consent to participate in the study before the procedures. All methods were carried out following relevant guidelines and regulations.

## Author Contributions

Study design, data analysis, and critical review: CB, CH, GL, LN, and LZ. Data analysis and critical review: PL. Data collection, data analysis, and critical review: AM, CB, JM, LB, LH, and SF. All authors contributed to the article and approved the submitted version.

## Funding

CH is a recipient of a scholarship of PUCPR and AFSM is recipient of a scholarship from CAPES. PL receives funding support from the National Heart, Lung, and Blood Institute (1R01HL134892), the American Heart Association (18CSA34080399), the RRM Charitable Fund, and the Simard Fund.

## Conflict of Interest

PL is an unpaid consultant to, or involved in clinical trials for Amgen, AstraZeneca, Baim Institute, Beren Therapeutics, Cartesian, Esperion, Therapeutics, Genentech, Kancera, Kowa Pharmaceuticals, Medimmune, Merck, Novo Nordisk, Merck, Novartis, Pfizer, Sanofi-Regeneron. PL is a member of scientific advisory board for Amgen, Corvidia Therapeutics, Caristo, CSL Behring, DalCor Pharmaceuticals, Dewpoint, PlaqueTec, Kancera, Kowa Pharmaceuticals, Olatec Therapeutics, Medimmune, Novartis, and XBiotech, Inc.

PL’s laboratory has received research funding in the last 2 years from Novartis. PL is on the Board of Directors of XBiotech, Inc. PL has a financial interest in Xbiotech, a company developing therapeutic human antibodies. PL’s interests were reviewed and are managed by Brigham and Women's Hospital and Partners HealthCare in accordance with their conflict of interest policies.

The remaining authors declare that the research was conducted in the absence of any commercial or financial relationships that could be construed as a potential conflict of interest.

## Publisher’s Note

All claims expressed in this article are solely those of the authors and do not necessarily represent those of their affiliated organizations, or those of the publisher, the editors and the reviewers. Any product that may be evaluated in this article, or claim that may be made by its manufacturer, is not guaranteed or endorsed by the publisher.

## Supplementary Material

Supplementary Material Photomicrograph demonstrating the positive and negative controls of the biomarkers studied (CD163, Casp-1, Casp-9, GSDM-D, ICAM-1, TNF-alpha, MMP-9, IL-4, IL-6, IL-1beta, TGF -beta, TUNEL). Placenta with CD163 positive histiocytes (arrow), Casp-1 and Casp-9 positive skeletal muscle fibers (arrow), GSDM-D positive enterocytes (arrow), ICAM-1 positive pulmonary capillaries (arrow) can be seen; cutaneous inflammatory process positive for TNF-alpha, IL-4, IL-6, and TGF-beta (arrow) and fibrous alveolar septum positive for MMP-9 and IL-1beta (arrow) can also be observed.
