# Supplementary figures and images for: The Pathogenesis of COVID-19 Myocardial Injury: An Immunohistochemical Study of Postmortem Biopsies
Source: Front Immunol. 2021 Nov 5;12:748417. doi: 10.3389/fimmu.2021.748417 (PMC8602833; doi:10.3389/fimmu.2021.748417)

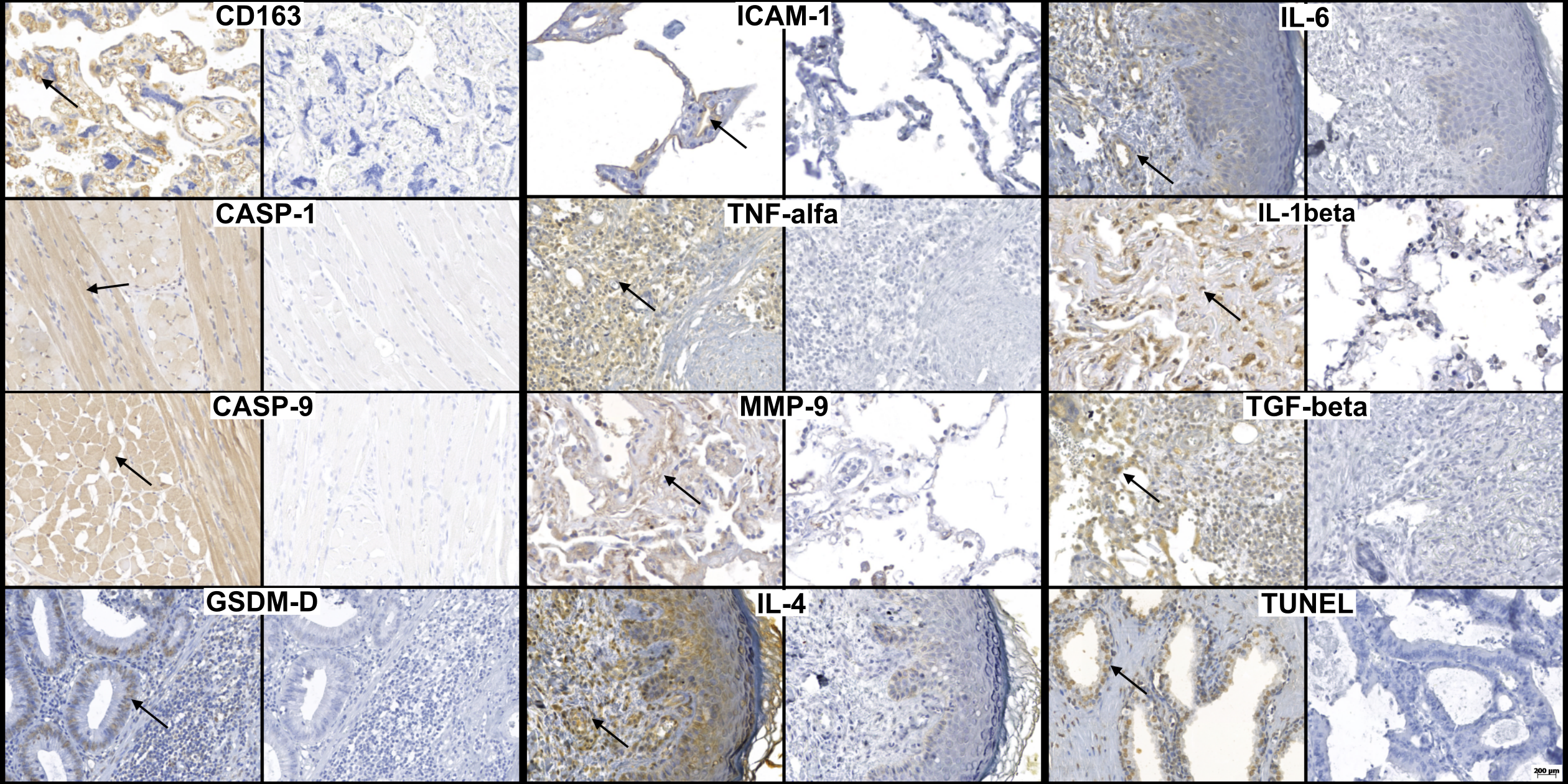

Supplement: Supplementary Material — Photomicrograph demonstrating the positive and negative controls of the biomarkers studied (CD163, Casp-1, Casp-9, GSDM-D, ICAM-1, TNF-alpha, MMP-9, IL-4, IL-6, IL-1beta, TGF -beta, TUNEL). Placenta with CD163 positive histiocytes (arrow), Casp-1 and Casp-9 positive skeletal muscle fibers (arrow), GSDM-D positive enterocytes (arrow), ICAM-1 positive pulmonary capillaries (arrow) can be seen; cutaneous inflammatory process positive for TNF-alpha, IL-4, IL-6, and TGF-beta (arrow) and fibrous alveolar septum positive for MMP-9 and IL-1beta (arrow) can also be observed. [file Image_1.tiff]
