# Supplementary material for: The Pathogenesis of COVID-19 Myocardial Injury: An Immunohistochemical Study of Postmortem Biopsies
Source: Front Immunol. 2021 Nov 5;12:748417. doi: 10.3389/fimmu.2021.748417 (PMC8602833; doi:10.3389/fimmu.2021.748417)
Supplement: Supplementary file 2 [file Table_1.docx]

**Supplementary material 1** – Specifications of the antibodies used to investigate the formalin-fixed, paraffin-embedded myocardial tissues

| **Antibody** | **Type** | **Clone/Code** | **Dilution** | **Source** |
| --- | --- | --- | --- | --- |
| Anti-Casp 1 | Polyclonal/Rabbit | ab189796 | 1:200 | Abcam |
| Anti-Casp 9 | Polyclonal/Rabbit | PAA627Hu01 | 1:100 | Cloud Clone |
| Anti-CD163 | Monoclonal/Mouse | Ab9324 | 1:400 | Abcam |
| Anti-Col 1 | Polyclonal/Rabbit | ab34710 | 1:200 | Abcam |
| Anti-Col 3 | Polyclonal/Rabbit | Ab 7778 | 1:200 | Abcam |
| Anti-GSMD | Polyclonal/Rabbit | PA5-104324 | 1:50 | Thermo Fisher |
| Anti-ICAM 1 | Monoclonal/Mouse | 23G12 | 1:100 | Novocastra |
| Anti-IL-4 | Polyclonal/Rabbit | PA525165 | 1:200 | Thermo Fisher |
| Anti-IL-6 | Monoclonal/Mouse | Ab9324 | 1:400 | Abcam |
| Anti-IL-1β | Polyclonal/Rabbit | A16288 | 1:800 | ABclonal |
| Anti-MMP 9 | Monoclonal/Mouse | EP1254 | 1:200 | Abcam |
| Anti-TGF-β | Polyclonal/Rabbit | E11262 | 1:200 | Spring |
| Anti-TNF-α | Polyclonal/Rabbit | Ab6671 | 1:100 | Abcam |
| TUNNEL assay | In Situ Cell Death Detection Kit, POD (Roche, Mannheim, Germany) | | | |
