# Supplementary material for: The Pathogenesis of COVID-19 Myocardial Injury: An Immunohistochemical Study of Postmortem Biopsies
Source: Front Immunol. 2021 Nov 5;12:748417. doi: 10.3389/fimmu.2021.748417 (PMC8602833; doi:10.3389/fimmu.2021.748417)
Supplement: Supplementary file 3 [file Table_2.docx]

**Supplementary material 2** – Detailed clinical data from the baseline of COVID-19 patients

|  | Patient 1 | Patient 2 | Patient 3 | Patient 4 | Patient 5 | Patient 6 |
| --- | --- | --- | --- | --- | --- | --- |
| Gender, Age (years) | Male, 73 | Male, 80 | Male, 57 | Female, 81 | Male, 75 | Male, 70 |
| Underlying Conditions | Type 2 Diabetes Mellitus Chronic Kidney Disease  Atrial Fibrillation Coronary Artery Disease Heart Failure Peripheral Obstructive Artery Disease | Arterial Hypertension Coronary Artery Disease Class III obesity | Type 2 Diabetes Mellitus Arterial Hypertension Coronary Artery Disease  Hepatic Steatosis | Type 2 Diabetes Mellitus Arterial Hypertension  Dyslipidemia  Heart Failure | Type 2 Diabetes Mellitus Arterial Hypertension  Dyslipidemia  Hyperuricemia  Coronary Artery Disease Myocardial infarction (April-2020) | Type 2 Diabetes Mellitus Arterial Hypertension  Atrial Fibrillation  Interstitial Pulmonary Fibrosis  Pulmonary Hypertension  Former smoker  Heart Failure |
| Medications | Acetylsalicylic Acid Clopidogrel Rosuvastatin Losartan Hydrochlorothiazide Metoprolol Insulin NPH Cilostazol Erythropoietin | Acetylsalicylic Acid  Metoprolol Rivaroxaban  Ezetimibe  Pitavastatin  Trimetazidine  Carbamazepine  Trazodone 150  Inhaled Beclomethasone  Inhaled Formoterol 12 | Acetylsalicylic Acid  Rosuvastatin  Losartan  Metformin  Bupropion | Acetylsalicylic  Enalapril  Atorvastatin  Bisoprolol  Metformin  Glibenclamide  Empagliflozin  Linagliptin  Sodium Alendronate | Acetylsalicylic Acid  Clopidogrel  Candesartan  Levanlodipine  Chlortalidone  Atenolol  Atorvastatin  Metformin  Dapagliflozin  Glimepiride  Alogliptin  Pioglitazone | Ramipril  Metoprolol  Rosuvastatin  Warfarin 2.5mg  Glicazide  Dapagliflozin  Linagliptin  Duloxetine |
| Length of stay on Mechanical Ventilation | 10 days | 21 days | 9 days | 14 days | 9 days | 15 days |
| Chest Computed tomography at admission | Diffuse and bilateral “opacities with ground-glass attenuation”, suggestive of viral pulmonary infection | Diffuse and bilateral “opacities with ground-glass attenuation”, suggestive of viral pulmonary infection | Peripheral, multifocal and bilateral “opacities with groundglass attenuation”, suggestive of viral pulmonary infection. Presence of bronchial thickening. | Diffuse and bilateral “opacities with groundglass attenuation”, thickening of the pulmonary septum, suggestive of viral pulmonary infection. | Diffuse and bilateral “opacities with groundglass attenuation”, thickening of the pulmonary septum, suggestive of viral pulmonary infection. Presence of bronchial thickening. Presence of diffuse bilateral bronchiectasis. Presence of parasseptal emphysema. | Peripheral, multifocal and bilateral “opacities with groundglass attenuation”, suggestive of viral pulmonary infection. Interstitial Pulmonary Fibrosis. Cardiomegaly. Increased Pulmonary Artery Diameter (32mm). |
| Relevant initial laboratory tests | C-Reactive Protein = 83 mg/dL D-dimer = 3436 µg/mL  hs-Troponin I = 12,6 pg/mL  Creatinine = 7.45 mg/dL  Globular volume = 25%  Hemoglobin = 8.6 g/dL Leukocytes = 9,200 | C-Reactive Protein = 52 mg/dL D-dimer = 816 µg/mL  hs-Troponin I = 10.9 pg/dL  Creatinine = 0.74 mg/dL  Globular volume = 37.5%  Hemoglobin = 12.8 g/dL  Leukocytes = 4,700 | C-Reactive Protein = 154.2 mg /dL  D-dimer = 628 µg/mL  hs-Troponin I = 3.9 pg/mL  Creatinine = 0.82 mg/dL Globular volume = 38.3%  Hemoglobin = 14g/dL Leukocytes = 14,500 | C-Reactive Protein = 199.4 mg /dL  D-dimer = 83,143 µg/mL  hs-Troponin I = 42.1 pg/mL  Creatinine = 1.33 mg/dL Globular volume = 41.3%  Hemoglobin = 13.7 g/dL Leukocytes = 22,100 | C-Reactive Protein = 267mg/dL  D-dimer = 152,174 µg/mL  hs-Troponin I = 13.3pg/mL  Creatinine = 1.61 mg/dL Globular volume = 43.1%  Hemoglobin = 14.9 g/dL  Leukocytes = 13,100 | C-Reactive Protein = 156.9 mg/dL  D-dimer = 1,848 µg/mL  hs-Troponin I = 1750.2 pg/mL  Creatinine = 1.37 mg/dL Globular volume = 52.8%  Hemoglobin = 18.2 g/dL Leukocytes = 16,000 |
| Laboratory tests 24 hours before death | C-Reactive protein = 270 mg/dL  D-dimer = 4,858 µg/mL  hs-Troponin I = 87.4 pg/dL  Creatinine = 5.08 mg/dL Globular volume = 23%  Hemoglobin = 8.0 g/dL Leukocytes = 22,000 | C-Reactive protein = 407 mg/dL D-dimer = 4,507 µg/mL  hs-Troponin I = 32.7 pg/dL  Creatinine = 1.81 mg/dL Globular volume = 29.4%  Hemoglobin = 9.7 g/dL Leukocytes = 9,400 | C-Reactive Protein = 267.2 mg/dL  D-dimer = 6,571 µg/mL hs-Troponin I = 19.9 pg/mL Creatinine = 2.43 mg/dL  Globular volume = 27%  Hemoglobin = 9 g/dL  Leukocytes = 15,300 | C-Reactive Protein = 16.3 mg/dL  D-dimer = 19,137 µg/mL hs-Troponin I = 324,7 pg/mL Creatinine = 1.06 mg/dL  Globular volume = 29.2%  Hemoglobin = 9.5 g/dL  Leukocytes = 28,900 | C-Reactive Protein = 226.8 mg/dL  Troponina = 21,2 µg/mL Creatinine = 2.14 mg/dL  Globular volume = 19.7%  Hemoglobin = 11 g/dL Leukocytes = 19,100 | C-Reactive Protein = 8.7 mg/dL  Troponina = 245,2 pg/mL Creatinine = 1.66 mg/dL  Globular volume = 32%  Hemoglobin = 11.3 g/dL Leukocytes = 10,500 |
| Echocardiogram 24 hours before death | Ejection fraction = 43%  Left ventricle = mild eccentric hypertrophy; akinesia of the infero-lateral and basal lower walls.  Right ventricle = increased basal dimension and normal systolic function. sPAP = 68 mmHg. | Ejection fraction = 65%  Left ventricle = preserved dimensions.  Right ventricle = preserved dimensions and normal systolic function. sPAP = normal. | Ejection fraction = 64%  Left ventricle = preserved dimensions.  Right ventricle = Increased dimensions and slightly reduced systolic function. sPAP = 51 mmHg. | Ejection fraction = 45%.  Left ventricle = mild eccentric hypertrophy; hypokinesia of the lower-basal and inferoseptal walls.  Right ventricle = preserved dimensions and normal systolic function. sPAP = 34 mmHg. | Ejection fraction = 66%  Left ventricle = preserved dimensions.  Right ventricle = preserved dimensions and normal systolic function. sPAP = 28 mmHg. | Ejection fraction = 57%  Left ventricle = severe eccentric hypertrophy.  Right ventricle = Increased dimensions and compromised systolic function. sPAP = 70 mmHg. |
| Therapeutic drugs | Hydroxychloroquine  Azithromycin  Oseltamivir  Metronidazole  Meropenem  Linezolid | Hydroxychloroquine  Azithromycin  Oseltamivir  Ceftriaxone | Azithromycin  Ceftriaxone  Dexamethasone  Enoxaparin (prophylactic)  Piperacillin + Tazobactam  Alteplase for thrombolysis of PE | Azithromycin  Ceftriaxone  Oseltamivir  Dexamethasone  Enoxaparin  Piperacillin + Tazobactam | Azithromycin  Ceftriaxone  Dexamethasone  Enoxaparin (full) | Ceftriaxone  Azithromycin  Tocilizumabe  Methylprednisolone  Piperacillin + Tazobactam Enoxaparin (full) |
| Invasive procedure | Hemodialysis 3 times a week | Tracheostomy | Chemical Thrombolysis | Tracheostomy | Chest Tube Right (pneumothorax) | Tracheostomy |

* Reference values: hs-Troponin I < 19,8 pg/mL, D-dimer < 500 µg/mL. The choice of the antibiotics was done according to the diagnosis and protocol for the patient's profile. sPAP = systolic pressure in pulmonary artery.
