# Supplementary material for: The Pathogenesis of COVID-19 Myocardial Injury: An Immunohistochemical Study of Postmortem Biopsies
Source: Front Immunol. 2021 Nov 5;12:748417. doi: 10.3389/fimmu.2021.748417 (PMC8602833; doi:10.3389/fimmu.2021.748417)
Supplement: Supplementary file 4 [file Table_3.docx]

**Supplementary material 3** – Scores of biomarker expression in the myocardium samples according to the histological and immunohistochemical analysis.

| Patient | H&E | Toluidine Blue | CD163 | TUNEL assay | casp1 | casp9 | ICAM-1 | IL-1 β | IL-4 | IL-6 | MMP-9 | TNF-α | TGF-β | col1 | col3 | GSDM-D |
| --- | --- | --- | --- | --- | --- | --- | --- | --- | --- | --- | --- | --- | --- | --- | --- | --- |
| COVID19 (1) | edema; neutrophilic myocarditis | 1.25 | 6,89 | 5+3 | 5+3 | 4+2 | 4+3 | 4+3 | 5+3 | 5+3 | 5+3 | 3+2 | 1+1 | 5+2 | 5+3 | 4+3 |
| Control 1.1 | hypertrophy; infarction | 0.05 | 0.89 | - | 1+1 | 4+2 | 1+1 | 2+2 | 1+1 | 1+1 | 1+1 | 1+1 | 1+1 | 3+3 | 3+3 | 2+3 |
| COVID19 (2) | edema | 0.5 | 7.44 | 5+3 | 5+2 | 4+2 | 5+2 | 4+3 | 5+3 | 5+3 | 5+3 | 4+1 | 2+1 | 5+2 | 5+3 | 4+3 |
| Control 2.1 | cardiomyosclerosis | 0.05 | 4.67 | - | 1+1 | 4+2 | 1+1 | 2+2 | 2+2 | 4+3 | 1+1 | 1+1 | 1+1 | 3+3 | 2+3 | 4+3 |
| Control 2.2 | miocardium fibrosis | 0.6 | 2.11 | - | 2+2 | 4+3 | 1+1 | 2+1 | 3+2 | 4+3 | 1+1 | 1+1 | 1+1 | 2+3 | 2+3 | 4+3 |
| COVID19 (3) | edema | 0.45 | 4.4 | 5+3 | 4+2 | 4+2 | 5+3 | 3+3 | 5+3 | 5+3 | 4+2 | 5+3 | 1+1 | 5+3 | 5+3 | 3+2 |
| Control 3.1 | hypertrophy | 0.1 | 1.22 | - | 2+3 | 4+2 | 1+1 | 2+2 | 2+2 | 4+3 | 2+2 | 1+1 | 1+1 | 2+3 | 2+3 | 4+3 |
| Control 3.2 | hypertrophy | 0.05 | 3.44 | - | 1+1 | 4+2 | 1+1 | 2+1 | 1+1 | 1+1 | 1+1 | 1+1 | 1+1 | 2+3 | 2+3 | 4+3 |
| COVID19 (4) | edema | 0.55 | 7.7 | 5+3 | 3+2 | 4+2 | 4+2 | 4+3 | 5+3 | 5+3 | 4+1 | 5+3 | 2+1 | 5+3 | 5+3 | 2+2 |
| Control 4.1 | coronary disease | 0.15 | 3.11 | - | 1+2 | 4+2 | 1+1 | 2+2 | 1+1 | 3+3 | 2+2 | 1+1 | 1+1 | 2+3 | 2+3 | 4+3 |
| Control 4.2 | hypertrophy | 0.05 | 7.44 | - | 2+3 | 4+2 | 1+1 | 2+2 | 1+3 | 1+1 | 1+1 | 1+1 | 1+1 | 2+3 | 1+3 | 4+3 |
| COVID19 (5) | edema | 0.2 | 10.8 | 5+3 | 3+2 | 4+3 | 5+3 | 4+3 | 5+3 | 5+3 | 4+2 | 5+3 | 3+2 | 5+3 | 5+3 | 4+3 |
| Control 5.1 | coronary disease | 0.1 | 3.67 | - | 1+2 | 4+2 | 1+1 | 2+2 | 2+2 | 2+1 | 2+2 | 1+1 | 1+1 | 3+3 | 2+3 | 4+3 |
| Control 5.2 | hypertrophy | 0.15 | 1.67 | - | 1+2 | 4+2 | 1+1 | 1+1 | 2+2 | 3+3 | 1+1 | 1+1 | 1+1 | 2+3 | 2+3 | 4+3 |
| COVID19 (6) | edema | 0.4 | 7.86 | 5+3 | 4+3 | 4+2 | 5+2 | 4+2 | 5+3 | 5+3 | 4+3 | 5+3 | 2+2 | 5+3 | 5+3 | 2+2 |
| Control 6.1 | hypertrophy | 0.05 | 5.78 | - | 1+1 | 4+2 | 2+2 | 2+1 | 2+2 | 3+3 | 1+1 | 1+1 | 1+1 | 3+3 | 2+3 | 5+3 |
| Control 6.2 | hypertrophy; infarction | 0.05 | 3.56 | - | 1+2 | 4+2 | 1+1 | 2+2 | 1+1 | 2+2 | 2+3 | 1+1 | 1+1 | 2+3 | 2+3 | 4+3 |
| pvalue | - | 0.0023 | 0.0021 | - | 0.0010 | >0.9999 | <0.0001 | <0.0001 | <0.0001 | <0.0001 | 0.0002 | <0.0001 | 0.0063 | <0.0001 | <0.0001 | 0.0419 |

* Toluidine Blue and CD163: the count of mast cells degranulating per 20 high-power field under the microscope.

* * CD163: the count of histiocyte per 20 high-power field under the microscope.
